# Non-systemic treatment of metastatic kidney cancer: systematic review and a case report

**DOI:** 10.3389/fonc.2025.1627467

**Published:** 2025-12-12

**Authors:** Gabija O. Morkunaite, Ugne Mickeviciute, Mantas Trakymas, Marius Kincius, Vincas Urbonas, Giedrius Kvederas, Ausvydas Patasius

**Affiliations:** 1 Faculty of Medicine, Lithuanian University of Health Sciences, Kaunas, Lithuania; 2 Life Sciences Centre, Vilnius University, Vilnius, Lithuania; 3 Centre of Clinical Research, National Cancer Institute, Vilnius, Lithuania; 4 Centre of Orthopedics and Traumatology, Vilnius University, Faculty of Medicine, Institute of Clinical Medicine, Clinic of Rheumatology, Orthopaedic Traumatology and Reconstructive Surgery, Vilnius University Hospital, Santaros Clinics, Vilnius, Lithuania; 5 Laboratory of Cancer Epidemiology, National Cancer Institute, Vilnius, Lithuania; 6 Faculty of Medicine, Institute of Health Sciences, Vilnius University, Vilnius, Lithuania

**Keywords:** cryoablation, local treatment, metastasectomy, metastatic kidney cancer, RCC, SBRT

## Abstract

**Background and objectives:**

To date, there is insufficient literature on the local treatment options for metastatic renal cell carcinoma (mRCC). Various options, such as metastasectomy, thermal ablation, and stereotactic body radiotherapy, have gained attention for their potential to improve survival and quality of life in selected patients’ groups. This systematic review analyzes the effectiveness of these approaches and highlights their potential to improve survival outcomes. By analyzing survival outcomes, this review aims to guide future research, thereby enhancing clinical decision-making.

**Materials and methods:**

A systematic review of studies published since 2009 was conducted, following PRISMA guidelines. Relevant articles were identified from PubMed and ScienceDirect databases, using search terms related to mRCC and non-systemic treatment. The review was not registered, and a protocol was not prepared. Additionally, a case report documenting the successful use of cryoablation in controlling metastases of kidney cancer was included to illustrate clinical relevance.

**Results:**

Thirteen studies met the inclusion criteria. Complete metastasectomy demonstrated improved overall survival (OS) and progression-free survival (PFS) compared to incomplete or no metastasectomy. Stereotactic body radiotherapy (SBRT) and other forms of radiotherapy provided effective palliation and durable disease control.

**Conclusions:**

This systematic review highlights the potential survival benefits for mRCC patients with the use of different local treatment modalities, especially in terms of complete metastasectomy. The findings show the need for continued research to optimize local treatment strategies, along with improving the quality of life for metastatic kidney cancer patients.

## 1 Introduction

As one of the most common malignancies within the urinary system, kidney cancer poses a significant impact worldwide. In 2020, there were over 430,000 new cases reported globally, with approximately 180,000 resulting in death (1). Among kidney cancers, clear cell renal cell carcinoma (ccRCC) stands out as the most common histological subtype, known to be resistant to chemotherapy and radiotherapy (2). Known for its tendency to metastasize and recur, ccRCCs account for 80% of malignant kidney tumors found in adults, of which ccRCC contributes to the majority of cancer related deaths (3, 4). Metastatic renal cell carcinoma (mRCC) is present at diagnosis in 20-30% cases at presentation, and an additional 20-30% progress to metastatic disease following initial treatment (5, 6). Even though metastases from RCC can arise at any anatomical location, they are the most common in lung, bone, liver and brain (7).

Metastasectomy is considered to be the standard local therapy for mRCC and it is frequently performed in order to treat advanced cancer, involving the surgical removal of metastases secondary to the primary tumor in other organs (8, 9). As per EAU guidelines, metastases are considered to be potentially curable only if all tumor sites are removed (10). According to NCCN guidelines, patients eligible for metastasectomy and nephrectomy include those with primary RCC and oligometastases, or those who develop oligometastases after a long disease-free interval (DFI) following nephrectomy (9). However, other local therapies, such as thermal ablation and SBRT, including novel treatment modalities, are also reported. Cryoablation has emerged as an effective alternative to invasive surgical methods, showing high efficacy in several studies (11). Focal therapy using cryoablation can preserve renal function, reduce bleeding risk and time, and shorten hospital stays. Nonetheless, current guidelines from the European Association of Urology (EAU) and American Urological Association (AUA) typically recommend partial nephrectomy for renal masses smaller than 4 cm, while advanced metastatic disease often necessitates additional approaches to control progression. Despite retrospective data indicating that complete resection of solitary or oligometastatic RCC is associated with a favorable prognosis regardless of race or geographic location, it still raises the question whether this is due to favorable tumor biology, metastasectomy, or both (12). Minimally invasive options are constantly evolving, but given its relatively recent application, the potential benefits of non-systemic treatment for mRCC remain a matter of debate (8). The less debated benefits of local treatment include the delay or discontinuation of systemic treatment, thereby avoiding associated toxicities (12).

In this review, non-systemic treatment refers to local therapeutic approaches that do not rely on systemic drug administration, in contrast to systemic therapies such as immunotherapy or molecularly targeted therapies. These non-systemic modalities, including metastasectomy, stereotactic radiotherapy, and thermal ablation, are directed at specific metastatic lesions rather than affecting the entire body, and may be particularly valuable in selected patient groups.

To date, there is insufficient literature on the local treatment options for metastatic kidney cancer, highlighting the need to explore potential advantages of these methods. This review seeks to compare different local therapies and their outcomes, in terms of overall survival, progression-free survival, quality of life, and adverse effects. By systematically collecting and analyzing current evidence, this systematic review intends to offer insights into the possible benefits and limitations of these treatments. Ultimately, the aim of the study is to evaluate the efficacy and safety of various local treatment methods for metastatic renal cell carcinoma, thereby enhancing clinical decision-making and guiding future research.

## 2 Materials and methods

### 2.1 Study design

**Figure 1:**
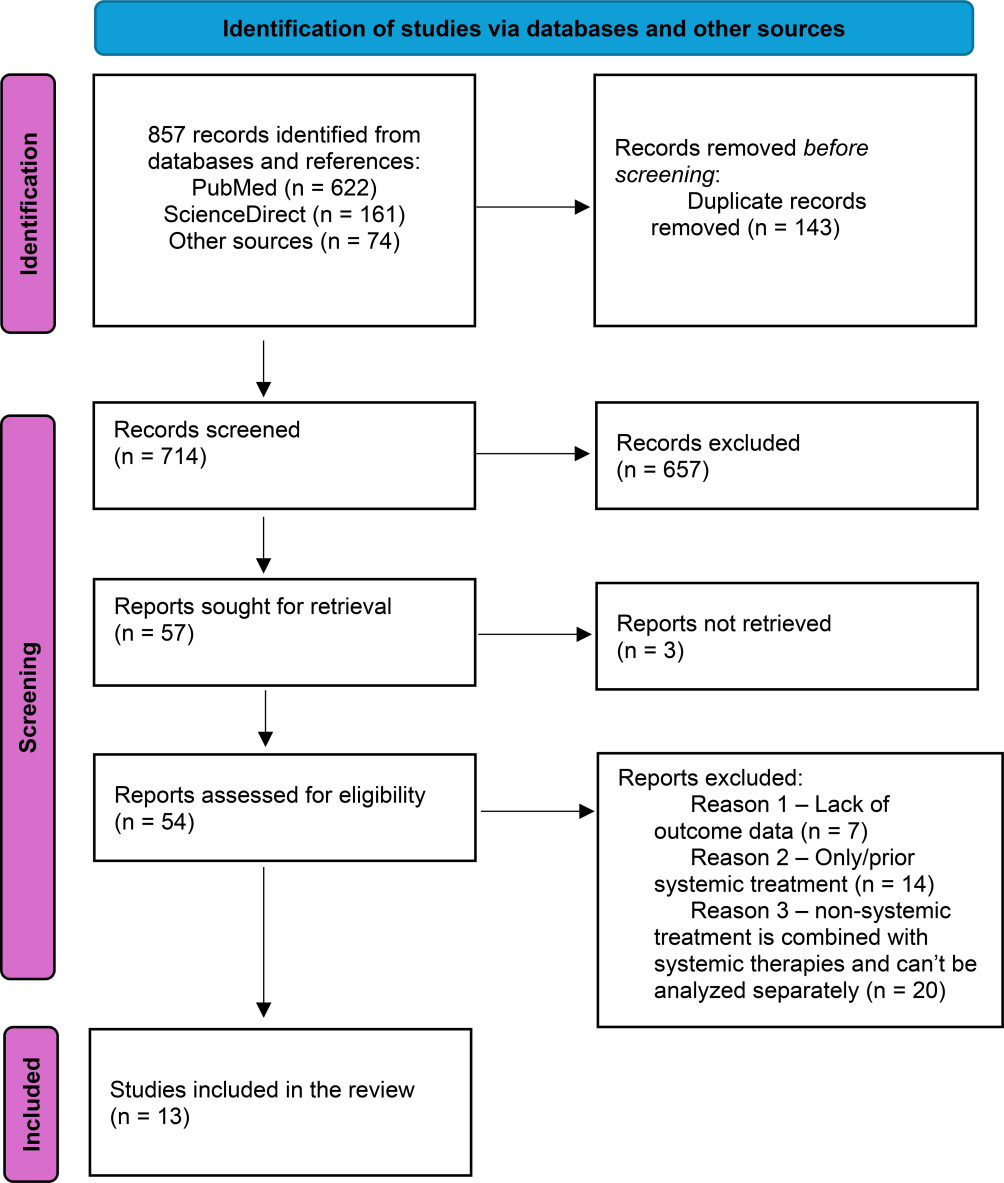
PRISMA flow diagram for identifying eligible studies.

A systematic review of literature was performed following the guidelines of the Preferred Reporting Items for Systematic Reviews and Meta-Analyses (PRISMA) guidelines (13). The review was not registered, and a protocol was not prepared. This article also incorporates a case report (**Supplementary Data**) relevant to the topic, to enhance the findings of the systematic review. The study selection process is summarized in the PRISMA flowchart (**Figure 1**).

### 2.2 Search strategy

To identify existing literature providing different local treatment options for metastatic renal cell carcinoma and their associated outcomes, a systematic search was conducted. We identified peer-reviewed studies published no earlier than 2009, January 1st by using the following search terms alone or combined: “renal cell carcinoma”, “metastatic”, “local”, “treatment”, “metastasectomy”, “cryotherapy”, “ablation”, “SBRT”. The terms were applied in the electronic databases PubMed and ScienceDirect, with filters used to narrow the search, followed by a manual review of reference lists for additional studies. PubMed and ScienceDirect were selected for their ability to provide a broad selection of studies pertinent to this review. Only articles written in English or containing an English abstract were eligible.

The search strategy included a range of terms, and their combinations related to local treatment modalities, including “SBRT”. Although “SBRT” was among the search terms, we did not limit the search to this term alone. During the screening phase studies, we used alternative terms such as stereotactic radiosurgery (SRS) and stereotactic radiotherapy (SRT), to ensure comprehensive coverage of these treatment techniques.

### 2.3 Eligibility criteria

Studies were considered if they included patients with metastatic renal cell carcinoma affecting any organ, except adrenal glands only. This criterion allowed inclusion of the studies that examined various metastatic sites, rather than those limited to the adrenal glands alone. Accepted interventions included metastasectomy, cryotherapy, thermal ablation, stereotactic radiosurgery, stereotactic body radiation therapy, whole brain radiotherapy, and image-guided radiation therapy. To be eligible, studies had to analyze local treatment either as a standalone approach or in combination with other non-systemic treatment modalities. Study types included retrospective, prospective studies, clinical trials and case reports.

### 2.4 Exclusion criteria

Studies were excluded if they focused on treatments for non-metastatic kidney cancer, if systemic treatments were either the sole treatment or were administered before non-systemic approaches, or if local treatments were combined with systemic therapies and couldn’t be analyzed separately. Additionally, studies focusing primarily on cytoreductive nephrectomy were excluded, as this intervention targets the primary tumor and falls outside the scope of our analysis, which focuses on local treatments directed at metastatic lesions. Studies lacking outcome data for non-systemic treatments, those using local therapies solely for palliative or analgesic purposes, and reviews, conference abstracts, letters, editorials or comments were also excluded. Studies involving children were not considered.

### 2.5 Data collection and analysis

Titles and abstracts of studies were imported into the reference manager software (Mendeley). Results from PubMed and ScienceDirect databases were manually screened, and the full text was retrieved as required.

### 2.6 Data extraction and management

Data were extracted from each identified paper, including details on the type of study, site of metastasis, treatment modality, and outcome measures. A descriptive review was performed.

Titles and abstracts of studies were imported into the reference manager software Mendeley. After screening the title list, abstracts were then reviewed for all relevant studies, and full texts were retrieved as required for all potentially relevant studies. Full papers and relevant abstracts were formally assessed to ensure they met the inclusion criteria for the systematic review. A peer-review process involving three authors was used to discuss studies with questionable eligibility for inclusion in the review. Data were extracted from each identified eligible paper. The extracted data included information on publication details (authors, title, year, and study design), trial design participants (age, sex, metastatic setting), type of treatment, and outcome measures. A descriptive review was conducted, and data were recorded in an Excel spreadsheet.

### 2.7 Quality assessment

The risk of bias for each included study was assessed independently by three authors based on seven categories:

1. Selection Bias;
2. Measurement of Confounders;
3. Confounding Adjustment;
4. Information Bias;
5. Treatment Details;
6. Sample Size Justification;
7. Reporting of Hypotheses.

Each category was assessed as having low, unknown, or high risk of bias using specific questions.

Selection bias was identified when inclusion and exclusion criteria were unclear or inconsistently applied.

Confounding was considered present if important baseline characteristics (e.g., patient demographics, disease stage, prior treatments) were either not reported, unevenly distributed between comparison groups, or not adjusted for in the analysis.

A study was classified as being at risk of information bias if outcomes such as overall survival or progression-free survival were not clearly defined, inconsistently measured, or if the measurement methods lacked standardization.

Treatment details were evaluated based on clarity and completeness in describing interventions such as metastasectomy or radiotherapy techniques.

A study was rated at risk in sample size justification if no rationale for sample size determination was provided.

Reporting of hypotheses was assessed by examining whether studies clearly stated their primary outcomes and research questions.

Any disagreements between reviewers were resolved through discussion and consensus.

## 3 Results

The PRISMA flowchart (**Figure 1**) displays a summary of studies identified through the literature search. A total of 857 records were identified, of which 714 were screened. 57 reports were selected for full-text screening, of which 3 studies could not be retrieved. Out of 54 reports, 13 studies were included in the systematic review after full text screening. The studies that did not meet the inclusion criteria were retained for discussion.

Out of the 13 included studies, 12 were retrospective, while only 1 study was prospective (**Table 1**). Six studies analyzed non-systemic treatment of metastases from renal cell carcinoma across various organs, with the most common sites being the lung, bone, liver, and brain. Less frequent sites in these studies included soft tissue, lymph nodes, adrenal glands, pancreas, kidney, and thyroid. The remaining seven studies focused more on one or two specific metastatic sites, emphasizing the spine (as part of bone involvement), lungs, pancreas, and liver.

**Table 1:** Characteristics of included studies.

Reference	Study design, country	Population	Age range, years	Sex, n (%)	Sites of metastasis	Type of treatment	Outcomes measured
*Lee et al (2023)* (14)	Retrospective, USA	mRCC treated with high-dose SBRT	59.2 ± 11.3	M: 45 (75) F: 15 (25)	Spine	SBRT	LC
*Zelefsky et al (2012)* (15)	Retrospective, USA	Extracranial metastatic lesions from primary RCC	–	M: 83 (79) F: 22 (21)	Bone	IGRT	LC, LR, 3-year LRFS, 1-year survival, 2-year survival, Median survival, Mean survival, SR
*Meyer et al (2018)* (21)	Retrospective, France	mRCC treated with SRT	37.5–87.6	M: 144 (76.6) F: 44 (23.4)	Brain, spine, viscera, non-spine bones, soft tissue	SRT	LRFS, PFS, TTS, OS, LC
*Sohn et al (2014)* (19)	Retrospective, South Korea	Spine metastasis from RCC treated with SRS or RT	SRS: 62.1 ± 9.6 RT: 25.0 ± 38.8	–	Spine	SRS, RT	OS, LC, PFS
*Dragomir, Nazha et al (2020)* (22)	Retrospective, Canada	Confirmed mRCC	*No-MSX:* 64 (57−72)	*No-MSX* M: 897 (73.5) F: 324 (26.5)	Lung, bones, adrenal glands, liver, brain, lymph nodes, pancreas, soft tissue, thyroid and other	MSX	OS, Risk of mortality
			*MSX:* 62 (55–68)	*MSX* M: 197 (78.8) F: 53 (21.2)			
*Sun et al (2018)* (23)	Retrospective, USA	Metastasectomy for mRCC	53–68	M: 4866 (69.6) F: 2128 (30.4)	Lung, liver, brain and other	MSX	OS
*Ranck et al (2013)* (24)	Retrospective, USA	mRCC	41–81	M: 11 (61.1) F: 7 (38.9)	Bone, abdominal lymph node, mediastinum/hilum, lung, kidney, adrenal glands, liver and soft tissue	SBRT	2-year PFS, Median DFS, Median time to ST, OS
*Nguyen et al (2010)* (16)	Prospective observational, USA	Spine metastasis from RCC	41–88	M: 36 F: 12	Spine	SBRT	PFS
*Maciolek et al (2018)* (25)	Retrospective, USA	mRCC	62–75 (IQR)	M: 15 (83) F: 3 (17)	Retroperitoneum, contralateral kidney, lung, adrenal gland, liver	Percutaneous microwave ablation	OS, RFS
*Kanzaki et al (2011)* (18)	Retrospective, Japan	Pulmonary metastasectomy from RCC	38–78	M: 38 F: 10	Lungs	MSX	OS
*Alt et al (2011)* (26)	Retrospective, USA	mRCC at the time of or after nephrectomy	*41–85 *(Yes)*	*M: 87 (69.6) F: 38 (30.4) *(Yes)*	Lungs, bone, viscera and other	MSX	CSS, OS
			21-92 *(No)*	M: 530 (69.5) F: 232 (30.5) *(No)*			
*Volk et al (2009)* (17)	Retrospective cohort, Germany	mRCC to the pancreas	54–74	M: 6 F: 8	Pancreas	MSX	CS
*Staehler (2010)* (20)	Retrospective comparative, Germany	mRCC to the liver	17-78	M: 57 (65) F: 30 (35)	Liver	MSX	5-year OS, Median survival

*Yes/No – patients who did and did not undergo complete surgical resection of multiple metastases. CSS, cancer-specific survival; CS, cumulative survival; DFS, disease-free survival; IQR, interquartile range; LC, local control; LR, local recurrence; LRFS, local recurrence-free survival; LPFS, local progression-free survival; MSX, metastasectomy; OS, overall survival; PFS, progression-free survival; RT, radiotherapy; SR, survival rate; SRT/SBRT, stereotactic (body) radiotherapy; ST, systemic therapy; TTS, time to systemic therapy.

The reporting and usage of systemic therapy varied across the included studies. Four studies did not provide information whether systemic therapy was used (14–17). One study did not give information on prior systemic therapy but mentioned that adjuvant chemotherapy was administered to some of the patients following metastasectomy (18). In one study, it is specified that stereotactic radiosurgery and external radiation therapy were applied exclusively as primary treatment choices (19). Another included study noted the use of systemic therapy in some patients in the later course of their disease, without providing specific details (20). Where systemic therapy was used, outcome data was available separately for those who did not receive prior systemic therapy, as seen in six studies (21–26). In one of those six studies, systemic therapies, including chemotherapy, immunotherapy, hormone therapy, and molecularly targeted therapies, were administered at various time points following diagnosis of metastatic disease (26).

### 3.1 Metastasectomy outcomes

Six studies evaluated the effectiveness of metastasectomy for renal cell carcinoma metastases, reporting outcomes of complete metastasectomy alone or in comparison to no-metastasectomy or incomplete metastasectomy (**Table 2**) (17, 18, 20, 22, 23, 26). Nevertheless, in one study, complete metastasectomy was performed in 90% of cases, with incomplete resections occurring due to reasons such as unresectable metastases (18). Complete metastasectomy was associated with significantly improved survival measurements in all six studies, including overall survival (OS), median survival, cumulative survival rates, and cancer-specific survival (CSC). These studies reported better outcomes for the complete metastasectomy groups in these various survival measures.

**Table 2:** Summarized results on the treatment outcomes across all the included studies.

Reference, (number of patients)	Notes	Treatment specifications	Results	p value
*Lee et al (2023)* (n=60) (14)	At 1 year after treatment, 44 treatment sites remained due to loss to follow-up or death. At 2 years, 15 patients were censored by death. No information on ST	SBRT Alone vs Separation Surgery* + SBRT * 23/75 spinal metastases in 60 patients	12 Month LC Rate: 87.3 % vs 89.5 % 24 Month LC Rate: 79.4 % vs 89.5 %	0.47
			LR: 6.6 ± 6.5 months 1-year survival: 62.2 % 2-year survival: 37.8 % Median survival: 9.1 months (range, 1.4–43.5) Mean survival: 12.1 ± 8.8 months. Cohort SR: 33.8 %	NR
*Zelefsky et al (2012)* (n=105) (15)	Phase I dose-escalation study was activated at 22 Gy. ST is not mentioned	Image-guided radiation therapy: High SD (n=45) vs Low SD (n=14) vs Hypofractionation (n=46)	3-year LRFS: 88% vs 21% vs 17%	0.001
			Improved LRFS in SD vs hypofractionation	0.01
			Improved LPFS in SD delivery of 24 Gy	0.01
*Meyer et al (2018)* (n=188) (21)	Oligoprogressive disease (n=101) Oligometastatic disease (n=80) After partial response to systemic therapy (n=7)	Stereotactic radiation therapy for 252 RCC metastases: Brain (n=20) Spine (n=75) Other (n=57)	LC at 6, 12 and 24 months: 87.5% vs 82.9% vs 77.6%	NR
			Median LRFS: 26.2 months (95% CI: 15.2–NR)	
			PFS: 7.6 months (95% CI: 5.8–13.4)	
			OS: 33.9 months (95% CI: 23.8–NR)	
			Median TTS: 14.2 months (95% CI: 10–27.2)	
			Median time to ST: 14.2 months (for all oligometastatic patients)	
			Residual disease PFS > Oligometastatic disease PFS	0.036
			More likely to have a better LRFS: oligoprogression	0.02
			Poorer LRFS with an increased number of systemic lines, and better for males.	0.035
				0.028
*Sohn et al (2014)* (n=26) (19)	Patients identified: SRS (n=13) RT (n=13) SRS or RT as a primary treatment only	Stereotactic radiosurgery (n=13) vs External radiation therapy (n=13)	Median OS: 15 vs 7 months	0.08
			LC at 1, 2, 3, 6 and 12 months: SRS 100 % vs 100 % vs 100 % vs 85.7 %, RT 100 % vs 91.7 % vs 83.3 % vs 58.3 % vs 29.2 %	NR
			PFS: SRS>RT	0.01
*Dragomir, Nazha et al (2020)* (n=1032) (22)	First-line targeted treatment prior to MSX or selection: 11.3% in the MSX group, 11.8% in the No-MSX group (p = 0.8477). Patients in the groups were matched	Complete metastasectomy (n=229) vs No metastasectomy (n=803)	OSR-5: 63.2 % vs 51.4 %	NR
			Risk of mortality MSX < No-MSX (hazard ratio: 0.41, 95% confidence interval: 0.27−0.63)	
			Targeted treatment during follow-up: 36.5 % vs 47.2 %	0.0026
			Median time to first-line targeted treatment during follow-up without prior ST: 49 months (IQR: 14−NR) vs 38 months (IQR: 11−66)	0.0057
*Sun et al (2018)* (n=6994) (23)	849 MSX patients received targeted therapy (43.0 %). No. of metastases is not specified	Metastasectomy (n=1976) vs No metastasectomy (n=5018)	1-, 2-, 5-year OS rates without targeted therapy: 68.7 %, 53.3 %, 27.1 % vs 61.8 %, 46.5 %, 23.7 % (n=1554)	0.004
*Ranck et al (2013)* (n=18) (24)	4 patients received prior ST. Separate analysis of patients who had treatment for all metastatic sites is available (none of them received prior ST)	Stereotactic body radiotherapy: Treatment for all sites (n=12)	Estimated 2-year PFS for all sites treated: 35.7 %	NR
			Median DFS for all sites treated: 12.7 months	
			Median time between SBRT to ST: 15.6 months	
			OS for all patients at 2 years: 85 %	
*Nguyen et al (2010)* (n=48) (16)	Prior radiation therapy (n=26). ST is not mentioned	Stereotactic body radiotherapy for 55 spinal metastases: Cervical (n=6) Thoracic (n=26) Lumbar (n=23)	1-year spine tumor PFS: 82.1 %	NR
*Maciolek et al (2018)* (n=18) (25)	4 patients (22 %) received prior ST prior to ablation which was continued after	Percutaneous microwave ablation for metastatic sites: Retroperitoneum (n=12) Contralateral kidney (n=6) Liver (n=6) Lung (n=5) Adrenal gland (n=5)	Median OS with prior ST vs no ST/ST after ablation: 2.0 vs 5.1 years	0.03
			2-year and 5-year RFS: 80 % (95 % CI 59–91 %)	NR
*Kanzaki et al (2011)* (n=48) (18)	8 patients underwent repeat resection, adjuvant chemotherapy was administered in 3 patients. Mostly solitary metastases, some with 2-4+	Pulmonary metastasectomy	5-year SR for complete vs incomplete resection: 50 % vs 20 %	0.034
			5-year SR for DFI ≥2 vs <2 years: 58 % vs 26 %	0.009
			5-year OS: 47 %	NR
*Alt et al (2011)* (n=887) (26)	In total, 404 patients (45.6%) received ST at various time points after they were diagnosed	Complete metastasectomy (n=125)	Median CSS for incomplete MSX with vs without ST: 1.6 vs 1.1 years	0.01
			Median CSS for complete MSX with vs without ST: 4.5 vs 5.7 years	0.98
			Median CSS for complete vs incomplete MSX: 4.8 vs 1.3 years	<0.001
			OS for complete vs incomplete MSX: 4.0 vs 1.3 years	<0.001
			Estimated 5-year CSS rate for complete vs incomplete MSX: 49.4 % vs 13.9 %	NR
			Estimated OS rate complete vs incomplete MSX: 44.5 % vs 12.9 %	
*Volk et al (2009)* (n=14) (17)	Not specified, if ST was given	Pancreatic metastasectomy	Median survival: 75 months (95% CI, 40.1–109.9)	NR
			CS for patients with metastasis <2.5 cm vs >2.5 cm: 100.9 vs 44.0 months	0.01
			CS for patients with solitary vs multiple metastasis: 111 vs 49 months	0.04
*Staehler (2010)* (n=88) (20)	20 patients served as a comparative group, since they declined surgery for liver metastasis. 79% MSX patients (n=54) received ST in the later course of their disease	Metastasectomy (n=68) vs No metastasectomy (n=20)	OSR-5 after MSX vs No MSX: 62.2 % ± 11.4 % (SEM) vs 29.3 % ± 22.0 % (SEM)	0.003
			Median survival after MSX vs No MSX: 142 months (95 % CI 115–169) vs 27 months (95% CI 16–38)	NR
			Median survival with metachronous metastases after MSX vs No MSX: 155 (95% CI 133–175) vs 29 months (95% CI 25–33)	0.001
			Median survival in low-grade primary RCC after MSX vs no MSX: 155 (95 % CI 123–187) vs 29 months	0.0036

CSS, cancer-specific survival; CS, cumulative survival; DFS, disease-free survival; DFI, disease-free interval; IQR, interquartile range; LC, local control; LR, local recurrence; LRFS, local recurrence-free survival; LPFS, local progression-free survival; MSX, metastasectomy; OS, overall survival; OSR-5, 5-year overall survival rate; PFS, progression-free survival; RT, radiotherapy; SBRT/SRT, stereotactic (body) radiotherapy; SEM, Standard Error of the Mean; SD, single dose; SR, survival rate; ST, systemic therapy; TTS, time to systemic therapy.

Two studies found that complete metastasectomy notably improved median OS (**Table 2**). One of the two studies revealed a significantly different median OS of 81 months (interquartile range [IQR]: 58−NR) compared to 61 months (IQR: 26−NR) between complete metastasectomy and no metastasectomy groups, respectively (p=0.0001) (22). Another study presented a median OS of 24.1 months versus 18.9 months (p<0.001). The 1-, 2-, 5-year OS rates of 68.4%, 50%, and 24.4% for the metastasectomy group, versus 64.1%, 43.0%, and 19.5% for the no metastasectomy groups (log-rank p<0.001) (27). One of the six studies also revealed significant relationships between disease-free intervals of less than 2 years versus 2 years or more, solitary versus multiple metastases, and completeness of resection (p<0.05). The 5-year survival rates were 50% and 20% for the complete and incomplete resection groups, respectively (18).

Another three studies specifically examined metastasectomy outcomes for metastases to lungs, liver and pancreas (**Table 2**). Pulmonary metastasectomy was associated with the cumulative survival rates of 60% at 3 years, 47% at 5 years, and 18% at 10 years (p=0.009). The majority of patients underwent resection of a solitary lung lesion, although some had 2, 3, or ≥4 resected (18). A study evaluating liver metastasectomy showed that the 5-year OS rate was 62.2% ± 11.4% (SEM), with a median survival of 142 months (95% CI: 115–169), which was significantly higher than the 29.3% ± 22.0% (SEM) and median survival of 27 months (95% CI: 16–38) seen in the no-metastasectomy group (p=0.003). In addition, metastasectomy of synchronous metastases did not significantly improve medial survival, which was 29 months (95% CI: 14-55) compared to 15 months (95%, CI 0-34) in the no metastasectomy group (p=0.593) (20). One study specifically focusing on pancreatic metastasectomy reported that patients with metastases smaller than 2.5 cm had a significantly longer survival after resection than those with metastases larger than 2.5 cm, (100.9 vs 44.0 months, respectively; p<0.01). Furthermore, a statistically significant difference was found when comparing survival between patients with solitary versus multiple metastasis, with survival of 111 months versus 49 months, respectively (p=0.04) (17). One study that focused on multiple metastatic sites also provided separate data on patients with lung-only metastases, revealing a 5-year CSS rate of 73.6% for complete metastasectomy group, compared to 19% for patients who did not undergo complete resection of all metastases (p<0.001) (26).

The use of systemic therapy and its impact varied across the studies (**Table 2**). In one study, a lower percentage of patients who underwent metastasectomy received targeted therapy during follow-up compared with those who did not (36.5% vs 47.2% respectively; p=0.0026), with a median time to first-line targeted treatment without prior exposure of 49 months and 38 months for the metastasectomy and no-metastasectomy groups, respectively (p=0.0057) (22). Another study demonstrated a survival benefit in patients treated with metastasectomy and targeted therapy (HR:0.86, 95% CI: 0.76–0.96, p=0.008) compared to those who received targeted therapy alone without metastasectomy. Additionally, a survival advantage was also found in patients who underwent metastasectomy without targeted therapy versus no-metastasectomy and no targeted therapy group (HR: 0.84, 95% CI: 0.75–0.94, p=0.004). The exact number of metastases per patient was not reported in the study, as the data captured metastatic sites only (23). One study pointed out that systemic treatment significantly improved survival only for patients who did not receive complete metastasectomy, showing a median CSS of 1.6 years for those who received systemic therapy versus 1.1 years for those who did not (p=0.01). Additionally, there was no significant difference observed in patients who underwent complete metastasectomy, with the median CSS being 4.5 years and 5.7 years with and without systemic therapy, respectively (p=0.98) (26).

### 3.2 Ablative and radiotherapy techniques

One of the included studies analyzed percutaneous microwave ablation as a treatment for metastatic renal cell carcinoma, reporting a 100% immediate technical success rate and durable local control for 93% of the tumors at a median follow-up of 14.7 months (25). However, the study also reported that patients with prior systemic therapy had an OS of 2.0 years, compared to 5.1 years for those who either did not receive systemic therapy or received it after the ablation procedure (p=0.03). This indicates that while percutaneous microwave ablation is effective for local control, the timing of systemic therapy is crucial in survival outcomes.

Image-guided radiotherapy (IGRT) was carried out in one study, using either a hypofractionated regimen or single dose (SD) irradiation (**Table 2**). The SD group had a significantly lower risk of local relapse, compared to the hypofractionation group (p=0.01). Also, in comparison with hypofractionation, SD treatment was associated with improved local relapse-free survival (p=0.01). To add on, a single dose of 24 Gy was a key predictor of enhanced local progression-free survival (PFS) (p=0.01) (15).

Three studies focused on the treatment of spine metastasis, using different radiotherapy techniques (**Table 2**). One study reported that the 1-year survival after stereotactic body radiotherapy (SBRT) was 62.2% and the 2-year survival was 37.8%. Among the patients who died, the median survival was 9.1 months (range, 1–43.4 months), and the mean survival was 12.1 ± 8.8 months. The most common regimen administered was a total dose of 27 Gy delivered in 3 fractions, followed by 40 Gy in 5 fractions and 18 Gy in 1 fraction, with regimens categorized as high-dose or low-dose using a BED (biological effective dose) of 50 Gy as a cutoff. There was no significant difference found in the rates of in-field progression between those with a BED > 50 Gy and those with a BED < 50 Gy (p=0.68). Also, the greatest pain reduction was seen in patients who initially reported moderate pain (4-6 out of 10) (14). Another study showed a 1-year spinal tumor progression-free survival rate of 82%. This study also reported that after SBRT, the percentage of pain-free patients increased to 44%, 53% and 64% at 1, 6 and 9 months, respectively, compared to 23% before treatment. No patient’s spinal cord received more than 45 Gy in total in this study (16). In the third spine study, outcomes of stereotactic radiosurgery (SRS) were reported in comparison to external radiation therapy (RT). The mean total margin radiation dose was 38.0 Gy for the SRS group and 29.4 Gy for the RT group (p=0.04), and mean nBED (normalized biological effective dose) was also significantly higher in the SRS group compared to RT (61.7 versus 32.0 Gy2/10, p=0.0001). The median overall survival was 15 months for the SRS group and 7 months for the RT group, but this difference was not statistically significant (p=0.08). When comparing perioperative Visual Analogue Scale (VAS) score changes, the SRS group demonstrated a larger decrease than the RT group (p=0.04) (19).

Another two studies focused on stereotactic radiotherapy (SRT) for multiple metastatic sites, including brain, spine, non-spine bones, soft tissue, viscera, lymph nodes, lung, kidney, adrenal gland, and liver (**Table 2**). One study performed SRT for oligoprogressive, oligometastatic, and residual tumors following a partial response to systemic treatment. For the entire cohort, median PFS was 8.5 months, local recurrence-free survival (LRFS) was 23.2 months, time to systemic therapy (TTS) was 13.2 months, and OS was 29.2 months. PFS was significantly longer in the residual tumor group compared to those with oligometastatic disease (p=0.036), while those with oligoprogression were more likely to have a better LRFS (local recurrence-free survival) (p=0.02). Also, LRFS was worse with an increased number of systemic treatments (p=0.035) and was higher in males than in females (p=0.0028) (21). In another study, multivariate analysis revealed that only an interval of less than 36 months from initial diagnosis to SBRT was a predictor of further metastatic progression (hazard ratio 2.37, p<0.001). Post SBRT, 50% of patients received systemic therapy, most commonly sunitinib, with a median time between SBRT and systemic therapy of 15.6 months. OS was estimated at 85% at two years, and no deaths occurred among patients who received treatment for all metastatic sites (24).

### 3.3 Assessment of quality

**Figure 2:**
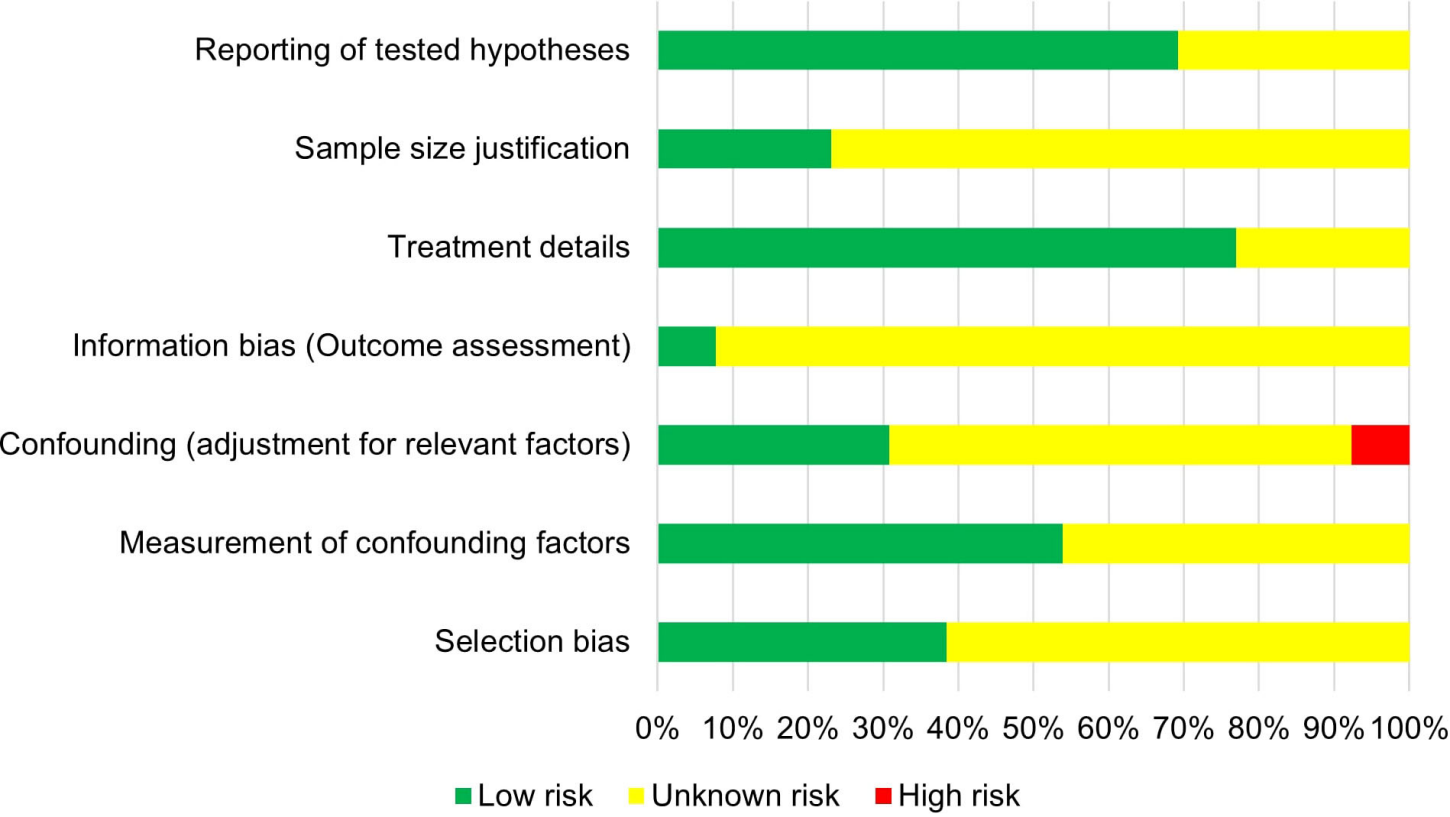
Risk of bias in various categories of included studies.

Risk of bias was assessed across seven categories, each of which contained two to three specific questions in order to evaluate potential bias. Three authors independently assessed each study. Based on evaluation, studies were classified as having low, unknown, or high risk of bias, as seen in **Figure 2**.

## 4 Discussion

Our systematic review indicates that specific mRCC patient subgroups can achieve substantial survival benefits from different local treatment approaches. In terms of survival measurements, all of them were consistently better for patients undergoing complete metastasectomy across the included studies, compared to those with incomplete or no metastasectomy (17, 18, 20, 22, 23, 26). Patients with solitary metastases, smaller lesions, or completeness of resection were also associated with the most favorable outcomes, suggesting that metastasectomy (MSX) may be particularly beneficial for these patient subgroups. The impact of systemic therapy additional to MSX was also noteworthy. While several studies demonstrated that adding systemic therapy after MSX carried additional survival benefits, one of the included studies found no significant improvement in OS for patients who received systemic therapy compared to those who did not. These mixed results indicate that the role of systemic therapy in conjunction with different types of local treatment is still unclear and needs further investigation.

We also analyzed other local treatment modalities, including different ablative techniques, such as percutaneous microwave ablation, and types of radiotherapy, including image-guided radiotherapy and stereotactic radiotherapy (14–16, 19, 21, 24, 25). Ablative therapies showed high technical success and durable local control of RCC, particularly for patients who have not received prior systemic therapy. However, the timing of systemic therapy was shown to be critical in some of the included studies. In terms of radiotherapy, SBRT showed promising results for various metastatic sites such as the spine, lung and other organs. For example, significant pain relief and prolonged PFS was seen in studies involving SBRT for patients with spine metastases, with an additional improvement in quality of life. This type of local treatment was also shown to give specific survival advantages for patients with multiple metastatic sites, with longer LRFS and PFS observed in patients treated with SBRT, particularly in those with oligoprogressive disease. Furthermore, although not included in our final review due to the use of systemic therapy, a retrospective Swedish study comparing SRT and surgical metastasectomy (SM) in patients with oligometastatic RCC provides valuable insight into survival outcomes (28). The study reported comparable OS between the two modalities, despite the fact that most of the patients were receiving targeted agents. These findings support the importance of local therapy, especially in selected patients with limited metastatic burden.

Although the original studies included in this systematic review used varying terms such as SBRT, SRT, and SRS, they all describe image-guided radiotherapy techniques delivering ablative doses in a limited number of fractions. For consistency, we use the term SBRT, unless explicitly referring to the term used by the original study. Additionally, to the studies included in our review, recent evidence supports the use of SBRT in metastatic RCC treatment.

The Delphi consensus developed by the European Society for Radiotherapy and Oncology (ESTRO), endorsed by the European Association of Urology, outlines recommendations for SBRT in both oligometastatic and oligoprogressive RCC. It emphasizes SBRT as a local treatment modality, particularly for patients with three to five metastatic lesions and controlled systemic disease, offering the potential to defer systemic therapy and improve quality of life (29). Furthermore, a comprehensive meta-analysis, involving 28 studies demonstrated high local control rates (more than 90%) and favorable survival outcomes following SBRT for oligometastatic RCC, reinforcing its role as an effective local treatment modality (30).

In recent years, the characterization of metastatic burden has become increasingly important in adapting local treatment methods for mRCC. Oligometastatic disease is defined as the presence of 1 to 5 metastatic lesions, according to the ESTRO-ASTRO consensus definition, with all sites potentially susceptible to effective local therapy (31).

The concept of oligoprogression, described in recent literature, refers to a situation where a limited number of metastatic lesions progress under systemic therapy. This clinical scenario allows for local treatment modalities to target progressing lesions while maintaining the current systemic therapy regimen (32).

In contrast, patients with multiple metastases are generally considered unsuitable for local treatment, and systemic therapy remains the recommended treatment approach according to ESMO Clinical Practice Guidelines (33).

These findings show that different local treatment modalities can provide substantial survival benefits when fitted to a specific patient. Once again, the impact of combining these interventions with systemic therapies remains different in each situation, with some studies showing improved outcomes and some of them showing no additional survival advantage with this type of combined treatment.

Tailoring local treatment strategies to individual patients is essential. Several factors may help identify patients who are most likely to benefit from non-systemic treatment approaches, as highlighted in the previous studies and consensus guidelines (29, 30). These include:

- Solitary or limited number of metastases (oligometastatic disease);
- Smaller metastatic lesion size;
- Longer disease-free interval before metastasis;
- Feasibility of achieving complete resection;
- Good performance status;
- Absence of brain or uncontrolled visceral metastases;
- No or delayed initiation of systemic therapy.

Considering these variables may support more individualized, evidence-based treatment decisions in clinical practice.

### 4.1 Strengths and weaknesses of the review

This systematic review provides important insights into the management of mRCC, especially by identifying different patient subgroups that benefit the most from specific local treatment methods. The main strength of this review is its comprehensive analysis and inclusion of various treatment modalities, including metastasectomy, various ablative therapies and different radiotherapy methods. Our findings highlight the significant survival advantages that specific patient subgroups can get, such as those with smaller lesions, or those undergoing complete metastasectomy, thereby showing the importance of personalized treatment strategies.

There are some limitations to our review. All included studies were of retrospective nature, apart from one prospective observational study, meaning the review relies on retrospective data. This highlights the need for more prospective studies to strengthen the evidence.

The retrospective nature of nearly all included studies introduces an inherent risk of bias. Retrospective analyses often involve selection bias, unmeasured confounding factors, and incomplete or inconsistent reporting of clinically relevant variables.

In addition, publication bias must be considered, as studies reporting positive or favorable outcomes for local treatment approaches are more likely to be published, while studies with negative or inconclusive findings may remain unpublished. This may result in an overestimation of the true effectiveness of these interventions across the available literature.

Most of the studies showed a low risk of bias in the treatment details and reporting hypothesis categories. However, there was a moderate risk of bias observed overall, with a significant number of included studies classified as having unclear risk of bias across various categories, such as selection bias, confounding adjustment, and information bias. Additionally, the conducted search was limited to studies published from 2009 onwards, and earlier publications might have been missed. For instance, the role of systemic therapy was not consistently addressed across all the included studies, including the unknown treatment timing (prior or after local treatment), potentially changing the survival outcomes. This inconsistency in systemic therapy timing limits our ability to isolate the true impact of local treatment and introduces potential interpretation bias when comparing survival outcomes across the studies. Furthermore, the included studies often lacked detailed reporting on clinically important factors such as the type and sequence of systemic therapy, time to progression, or the presence of brain or other specific metastases, limiting the possibility of performing subgroup comparisons. The inconsistency in reporting reduces the ability to fully explore which treatment conditions have the biggest impact on survival outcomes and represents a major limitation when applying findings to clinical practice.

### 4.2 The meaning of the results

The findings of our review suggest that specific local treatment modalities, including complete metastasectomy, advanced radiotherapy methods, and ablative techniques, play a significant role in treating mRCC. These local treatment techniques are seen to prolong survival outcomes and enhance the quality of life. However, it is important to note that specific patient and disease factors influence the benefits of local treatment strongly. This shows how complex the treatment for mRCC is highlights that local treatment is becoming more important. Furthermore, the review shows a significant lack of knowledge in understanding how local treatment modalities interact with systemic therapies, emphasizing the need for more evident work to create the best strategies.

### 4.3 Implications for practice, policy, and research

Findings of this systematic review highlight the importance of personalized treatment plans for each metastatic RCC patient. Clinicians should carefully consider different factors specific for each patient, such as the number, location and size of metastases, when selecting the type of local treatment. From a policy perspective, it is important to integrate local treatment options for mRCC into standard clinical practice. Future studies should focus on prospective designs to further investigate the role of local treatment in mRCC, particularly focusing on patient subgroups that could benefit the most. Additionally, it is important to explore local treatment in combination with systemic therapy, as the approach remains unclear.

### 4.4 Recommendations

Future research should focus on prospective studies to validate these retrospective findings, particularly analyzing the patient subgroups that respond best to different local interventions for metastatic kidney cancer. Exploration of combination of local treatment and systemic therapy should be done, with an emphasis on the timing. Guidelines should be developed in order to assist clinicians in adjusting treatment based on patient-specific characteristics.

## 5 Conclusions

This systematic review emphasizes the potential survival benefits of different local treatment modalities for metastatic renal cell carcinoma patients, especially in terms of complete metastasectomy, while highlighting the importance of individualized treatment plans. Even though the findings are mostly based on retrospective data, the need for prospective studies is apparent to validate this evidence. The mixed results regarding the use of systemic therapy in combination with local treatment highlight the importance of further investigation, with local treatments potentially serving as an alternative that could minimize the adverse effects associated with systemic therapy. Personalized treatment and multidisciplinary care are essential to improving survival and quality of life in patients with metastatic kidney cancer.

## Data Availability

The original contributions presented in the study are included in the article/**Supplementary Material**. Further inquiries can be directed to the corresponding author.
