# Supplementary material for: Non-systemic treatment of metastatic kidney cancer: systematic review and a case report
Source: Front Oncol. 2025 Dec 12;15:1627467. doi: 10.3389/fonc.2025.1627467 (PMC12740893; doi:10.3389/fonc.2025.1627467)
Supplement: Supplementary file 1 [file DataSheet1.docx]

Supplementary Data: Case Report

Non-systemic treatment of metastatic kidney cancer: systematic review and a case report

Gabija O. Morkunaite ^1,*^, Ugne Mickeviciute ^2,3^, Mantas Trakymas ^3^, Marius Kincius ^3^, Vincas Urbonas ^3^, Giedrius Kvederas ^4^ and Ausvydas Patasius ^5,6^

| Academic Editor: Firstname Lastname  Received: date  Revised: date  Accepted: date  Published: date  **Citation:** To be added by editorial staff during production.  **Copyright:** © 2025 by the authors. Submitted for possible open access publication under the terms and conditions of the Creative Commons Attribution (CC BY) license (https://creativecommons.org/licenses/by/4.0/). |
| --- |

^1^ Lithuanian University of Health Sciences, Faculty of Medicine, Kaunas, Lithuania;

^2^ Life Sciences Centre, Vilnius University, Vilnius, Lithuania;

^3^ Centre of Clinical Research, National Cancer Institute, Vilnius, Lithuania;

^4^ Centre of Orthopedics and Traumatology, Vilnius University, Faculty of Medicine, Institute of Clinical Medicine,

Clinic of Rheumatology, Orthopaedic Traumatology and Reconstructive Surgery, Vilnius University Hospital, San-

taros Clinics, Vilnius, Lithuania;

^5^ Laboratory of Cancer Epidemiology, National Cancer Institute, Vilnius, Lithuania;

^6^ Faculty of Medicine, Institute of Health Sciences, Vilnius University, Vilnius, Lithuania.

**^*^** Correspondence: gabija.mk@gmail.com

1. Case Presentation

A 53-year-old-woman was being monitored for a right kidney cyst and underwent a planned ultrasound scan, which revealed a 5 cm tumor. An abdominal computed tomography (CT) confirmed a right kidney medial pole tumor with abundant vascularization and infiltration of Gerota fascia (RENAL 2+2+3+x+3=10x). The tumor was managed with right transperitoneal laparoscopic nephrectomy at the time. The surgical pathology analysis revealed a ccRCC pT3aN0 G3 stage IV. Following the surgery, the patient was monitored closely. She was also diagnosed with scleroderma two years prior to nephrectomy.

Four years later, during the COVID-19 pandemic, the patient started experiencing pain in the middle third of the left humerus, in the left hip, and in the lower back and under the rib cage on the right side, and the severity of pain gradually increased. Performed MRI and CT suggested a possible metastasis in the left brachium area. Due to restrictions on healthcare facilities, the patient was only able to be examined after experiencing the pain for six months. Soon after, bone biopsy was performed, and pathology analysis confirmed the clinical diagnosis of ccRCC metastasis of the left humerus. The multidisciplinary team agreed on local treatment of the solitary metastasis, with a plan to start systemic treatment in case progression occurs.

A highly hypervascular tumor in the middle third of the humerus was revealed in the CT scan, which had destroyed the bone structure. A pathological humerus fracture with axial dislocation was also present. The decision was made to perform cryoablation of the tumor under CT guidance. A 7G IceRod cryo applicator was used to puncture the tumor, and two freeze-thaw cycles of 10 minutes each were performed. Due to the tumor hypervascular nature, a single applicator was insufficient, so additional 17G IceRod cryoapplicators were inserted through the muscle to puncture the tumor, and the same freeze-thaw cycles were repeated. 5 days later, the ablation was repeated on the areas which were insufficiently ablated. CT imaging confirmed an adequate ablative zone. Despite the unsuccessful embolization, cryoablation and osteosynthesis with an intramedullary nail were performed. According to the International Metastatic RCC Database Consortium (IMDC) classification, our patient was in the intermediate risk group. Due to the lack of other signs of systemic dissemination of disease, multidisciplinary team offered active surveillance. Follow-up CT scans at 3, 6, 9 months, 1 year, 1,5 years, 2 years post-cryoablation were negative for progression or recurrence, and the disease was stable.

At the age of 60, 2.5 years after cryoablation of left humerus metastasis, a CT scan revealed a 15 x 20 mm exo-endophytic tumor in the mid-lateral metastatic tumor in the mid-lateral region of the only remaining left kidney, located more than 7 mm away from the renal collecting system (cT1aN0M0). The postoperative period was uneventful, with the patient in satisfactory condition and creatinine levels within normal range. Two follow-up CT scans confirmed a stable disease, with the most recent at 9 months post-cryoablation.

**
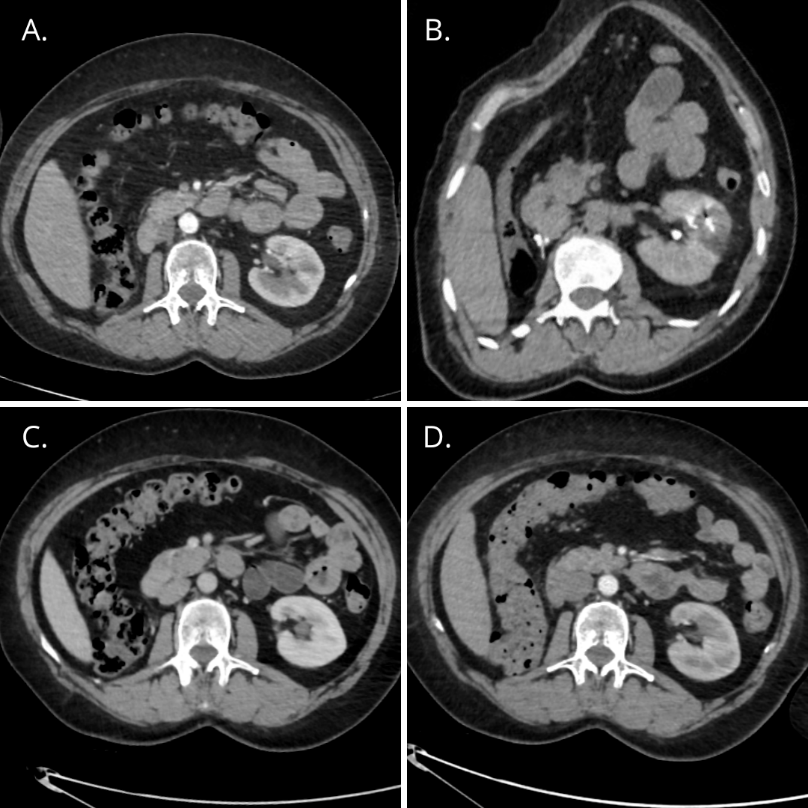
**

**Figure 3.** Solitary left kidney tumor (A), marked before cryoablation (B). Follow up CT scan at 4 months (C) and 9 months (D) reveal a stable disease.

**Author Contributions:** Conceptualization, A.P.; investigation, G.O.M.; formal analysis, G.O.M.; resources, M.T. and G.K.; visualization, G.O.M. and U.M.; writing—original draft preparation, G.O.M.; writing—review and editing, U.M., M.K., and V.U.; supervision, A.P.; project administration, A.P. All authors have read and agreed to the published version of the manuscript.

**Funding:** This research received no external funding.

**Informed Consent Statement:** Informed consent was obtained from the patient involved in the study. Written informed consent has been obtained from the patient to publish this paper.

**Data Availability Statement:** The original contributions presented in this study are included in the article material. Further inquiries can be directed to the corresponding author.

**Conflicts of Interest:** The authors declare no conflicts of interest.

References

1. Sung H, Ferlay J, Siegel RL, et al. Global Cancer Statistics 2020: GLOBOCAN Estimates of Incidence and Mortality Worldwide for 36 Cancers in 185 Countries. CA Cancer J Clin. 2021;71(3):209-249. doi:10.3322/caac.21660
2. Yang J, Wang K, Yang Z. Treatment strategies for clear cell renal cell carcinoma: Past, present and future. Front Oncol. 2023;13:1133832. Published 2023 Mar 21. doi:10.3389/fonc.2023.1133832
3. Qi X, Li Q, Che X, Wang Q, Wu G. The Uniqueness of Clear Cell Renal Cell Carcinoma: Summary of the Process and Abnormality of Glucose Metabolism and Lipid Metabolism in ccRCC. Front Oncol. 2021;11:727778. Published 2021 Sep 15. doi:10.3389/fonc.2021.727778
4. Hsieh JJ, Purdue MP, Signoretti S, et al. Renal cell carcinoma. Nat Rev Dis Primers. 2017;3:17009. Published 2017 Mar 9. doi:10.1038/nrdp.2017.9
5. Teishima J, Murata D, Inoue S, et al. Prediction of early progression of metastatic renal cell carcinoma treated with first-line tyrosine kinase inhibitor. Curr Urol. 2021;15(4):187-192. doi:10.1097/CU9.0000000000000042
6. Abreu D, Carvalhal G, Gueglio G, et al. Prognostic Factors in De Novo Metastatic Renal Cell Carcinoma: A Report From the Latin American Renal Cancer Group. JCO Glob Oncol. 2021;7:671-685. doi:10.1200/GO.20.00621
7. Vig SVL, Zan E, Kang SK. Imaging for Metastatic Renal Cell Carcinoma. Urol Clin North Am. 2020;47(3):281-291. doi:10.1016/j.ucl.2020.04.005
8. Dabestani S, Marconi L, Bex A. Metastasis therapies for renal cancer. Curr Opin Urol. 2016;26(6):566-572. doi:10.1097/MOU.0000000000000330
9. Matuszczak M, Kiljańczyk A, Salagierski M. Surgical Approach in Metastatic Renal Cell Carcinoma: A Literature Review. Cancers (Basel). 2023;15(6):1804. Published 2023 Mar 16. doi:10.3390/cancers15061804
10. Ljungberg B, Albiges L, Abu-Ghanem Y, et al. European Association of Urology Guidelines on Renal Cell Carcinoma: The 2022 Update. Eur Urol. 2022;82(4):399-410. doi:10.1016/j.eururo.2022.03.006
11. Green H, Taylor A, Khoo V. Beyond the Knife in Renal Cell Carcinoma: A Systematic Review-To Ablate or Not to Ablate?. Cancers (Basel). 2023;15(13):3455. Published 2023 Jun 30. doi:10.3390/cancers15133455
12. Dabestani S, Marconi L, Hofmann F, et al. Local treatments for metastases of renal cell carcinoma: a systematic review. Lancet Oncol. 2014;15(12):e549-e561. doi:10.1016/S1470-2045(14)70235-9
13. Hutton B, Salanti G, Caldwell DM, et al. The PRISMA extension statement for reporting of systematic reviews incorporating network meta-analyses of health care interventions: checklist and explanations. Ann Intern Med. 2015;162(11):777-784. doi:10.7326/M14-2385
14. Lee JH, Linzey JR, Strong MJ, et al. Local Control in Patients with Metastatic Renal Cell Carcinoma to the Spine: The Experience of an Institution with a Multidisciplinary Spine Oncology Program. World Neurosurg. 2023;178:e403-e409. doi:10.1016/j.wneu.2023.07.079
15. Zelefsky MJ, Greco C, Motzer R, et al. Tumor control outcomes after hypofractionated and single-dose stereotactic image-guided intensity-modulated radiotherapy for extracranial metastases from renal cell carcinoma. Int J Radiat Oncol Biol Phys. 2012;82(5):1744-1748. doi:10.1016/j.ijrobp.2011.02.040
16. Nguyen QN, Shiu AS, Rhines LD, et al. Management of spinal metastases from renal cell carcinoma using stereotactic body radiotherapy. Int J Radiat Oncol Biol Phys. 2010;76(4):1185-1192. doi:10.1016/j.ijrobp.2009.03.062
17. Volk A, Kersting S, Konopke R, et al. Surgical therapy of intrapancreatic metastasis from renal cell carcinoma. Pancreatology. 2009;9(4):392-397. doi:10.1159/000181174
18. Kanzaki R, Higashiyama M, Fujiwara A, et al. Long-term results of surgical resection for pulmonary metastasis from renal cell carcinoma: a 25-year single-institution experience. Eur J Cardiothorac Surg. 2011;39(2):167-172. doi:10.1016/j.ejcts.2010.05.021
19. Sohn S, Chung CK, Sohn MJ, et al. Stereotactic radiosurgery compared with external radiation therapy as a primary treatment in spine metastasis from renal cell carcinoma: a multicenter, matched-pair study. J Neurooncol. 2014;119(1):121-128. doi:10.1007/s11060-014-1455-9
20. Staehler MD, Kruse J, Haseke N, et al. Liver resection for metastatic disease prolongs survival in renal cell carcinoma: 12-year results from a retrospective comparative analysis. World J Urol. 2010;28(4):543-547. doi:10.1007/s00345-010-0560-4
21. Meyer E, Pasquier D, Bernadou G, et al. Stereotactic radiation therapy in the strategy of treatment of metastatic renal cell carcinoma: A study of the Getug group. Eur J Cancer. 2018;98:38-47. doi:10.1016/j.ejca.2018.04.008
22. Dragomir A, Nazha S, Wood LA, et al. Outcomes of complete metastasectomy in metastatic renal cell carcinoma patients: The Canadian Kidney Cancer information system experience. Urol Oncol. 2020;38(10):799.e1-799.e10. doi:10.1016/j.urolonc.2020.07.021
23. Sun M, Meyer CP, Karam JA, et al. Predictors, utilization patterns, and overall survival of patients undergoing metastasectomy for metastatic renal cell carcinoma in the era of targeted therapy. Eur J Surg Oncol. 2018;44(9):1439-1445. doi:10.1016/j.ejso.2018.05.026
24. Ranck MC, Golden DW, Corbin KS, et al. Stereotactic body radiotherapy for the treatment of oligometastatic renal cell carcinoma. Am J Clin Oncol. 2013;36(6):589-595. doi:10.1097/COC.0b013e31825d52b2
25. Maciolek KA, Abel EJ, Best SL, et al. Percutaneous microwave ablation for local control of metastatic renal cell carcinoma. Abdom Radiol (NY). 2018;43(9):2446-2454. doi:10.1007/s00261-018-1498-z
26. Alt AL, Boorjian SA, Lohse CM, Costello BA, Leibovich BC, Blute ML. Survival after complete surgical resection of multiple metastases from renal cell carcinoma. Cancer. 2011;117(13):2873-2882. doi:10.1002/cncr.25836
27. Kim SH, Park WS, Park B, Pak S, Chung J. A Retrospective Analysis of the Impact of Metastasectomy on Prognostic Survival According to Metastatic Organs in Patients With Metastatic Renal Cell Carcinoma. Front Oncol. 2019;9:413. Published 2019 May 22. doi:10.3389/fonc.2019.00413
